# Population transcriptogenomics highlights impaired metabolism and small population sizes in tree frogs living in the Chernobyl Exclusion Zone

**DOI:** 10.1186/s12915-023-01659-2

**Published:** 2023-07-31

**Authors:** Clément Car, André Gilles, Elen Goujon, Marie-Laure Delignette Muller, Luc Camoin, Sandrine Frelon, Pablo Burraco, Samuel Granjeaud, Emilie Baudelet, Stéphane Audebert, Germán Orizaola, Jean Armengaud, Arthur Tenenhaus, Imène Garali, Jean-Marc Bonzom, Olivier Armant

**Affiliations:** 1 Institut de Radioprotection Et de Sûreté Nucléaire (IRSN), PSE-ENV/SRTE/LECO, Cadarache, France; 2 PSE-SANTE/SESANE/LRTox, Fontenay Aux Roses, France; 3 UMR 1467 RECOVER, Aix-Marseille Université, INRAE, Centre Saint-Charles, Marseille, France; 4 Laboratoire Des Signaux Et Systèmes, Université Paris-Saclay, CNRS, CentraleSupélec, 91190 Gif-Sur-Yvette, France; 5 Laboratoire de Biométrie Et Biologie Evolutive, UMR 5558, Université de Lyon, Université Lyon 1, CNRS, VetAgro Sup, Villeurbanne, France; 6 Aix-Marseille University, Inserm, CNRS, Institut Paoli-Calmettes, CRCM, Marseille Proteomics, Marseille, France; 7 Animal Ecology, Department of Ecology and Genetics, Evolutionary Centre, Uppsala University, 75236 Uppsala, Sweden; 8 Doñana Biological Station (CSIC), Seville, Spain; 9 IMIB-Biodiversity Research Institute, University of Oviedo, 33600 Mieres-Asturias, Spain; 10 Zoology Unit, Department of Biology of Organisms and Systems, University of Oviedo, 33071 Oviedo-Asturias, Spain; 11 Département Médicaments Et Technologies Pour La Santé (DMTS), Université Paris-Saclay, CEA, INRAE, SPI, Bagnols-Sur-Cèze, France

**Keywords:** Genetics, Transcriptomics, Population, Amphibian, Pollution, Ionizing radiation, Chronic exposure, Modelling, SNP

## Abstract

**Background:**

Individual functional modifications shape the ability of wildlife populations to cope with anthropogenic environmental changes. But instead of adaptive response, human-altered environments can generate a succession of deleterious functional changes leading to the extinction of the population. To study how persistent anthropogenic changes impacted local species’ population status, we characterised population structure, genetic diversity and individual response of gene expression in the tree frog *Hyla orientalis* along a gradient of radioactive contamination around the Chernobyl nuclear power plant.

**Results:**

We detected lower effective population size in populations most exposed to ionizing radiation in the Chernobyl Exclusion Zone that is not compensated by migrations from surrounding areas. We also highlighted a decreased body condition of frogs living in the most contaminated area, a distinctive transcriptomics signature and stop-gained mutations in genes involved in energy metabolism. While the association with dose will remain correlational until further experiments, a body of evidence suggests the direct or indirect involvement of radiation exposure in these changes.

**Conclusions:**

Despite ongoing migration and lower total dose rates absorbed than at the time of the accident, our results demonstrate that *Hyla orientalis* specimens living in the Chernobyl Exclusion Zone are still undergoing deleterious changes, emphasizing the long-term impacts of the nuclear disaster.

**Supplementary Information:**

The online version contains supplementary material available at 10.1186/s12915-023-01659-2.

## Background

Rapid environmental change caused by human activities poses threats to many wild local species by increasing their vulnerability and notably increasing the risk of population extinction. Individual functional modifications of biological features (i.e. from gene to organism) determine this vulnerability [1]. Here, “function” is referring to a causal relationship between environmental trigger and individual response which impact the interaction between individuals and their environment [2]. These modifications can be detrimental, neutral or adaptive, as well as transmitted or non-transmitted. Functional adaptations in response to an altered environment have often been studied to disentangle plastic and selected processes [3–5]. But the interaction between evolutionary processes (irrespective of functional consequences, and adaptive nature) and plastic changes in the emergence of such functional modifications remains largely an open issue.

The current status of species and their ability to persist locally can be first explored by examining the evolutionary processes faced with contemporary environmental change [6]. By investigating population genetic diversity and structure, population genetics contributes towards disentangling the mechanisms that shape these evolutionary processes [7]. Short-term selection and changes of evolutionary potential in response to new abiotic and biotic interactions induced by human activities have often been emphasised for example in the case of urbanisation [8], climate change [9], or pollution [10]. This generally leads to a decrease in genetic diversity on standing variation or by selective sweeps. Other processes like migration can be also greatly modified by human activities and influence gene flow between populations, potentially increasing genetic diversity with population admixture [11] or decreasing genetic diversity by altering connectivity [12]. More broadly, in the case of small population size, random fixation of transmitted variations through genetic drift can lead to decrease in genetic diversity which can in turn affect population vulnerability [13, 14]. Also, anthropogenic abiotic changes like pollution can induce de novo mutations which could lead to an increased genetic diversity [15], thereby providing the source of genetic variability upon which evolution acts, but may have deleterious effects by directly impairing protein function [16, 17] or through indirect collateral effects [10, 18, 19]. In parallel with evolutionary processes, phenotypic plasticity interacts with individual functional changes and notably influences adaptive evolution. In small populations the increase of genetic drift limits the genetic potential on which selection can act and thus increases the importance of plastic changes to be able to cope with the altered environment [20]. On the other side, individual functional changes act also as feedback on evolutionary processes. For example, if plastic changes lead to a phenotype close to the fitness optimum, directional selection may only have a limited effect [20]. Combining the study of evolutionary processes (with or without direct individual functional consequences) with more functional-centred effects appears thus essential to estimate properly the vulnerability of populations facing anthropogenic pressures.

Pollutants constitute an interesting case study to analyse the evolutionary processes, phenotypic plasticity and functional responses of exposed organisms as they are examples of rapid and persistent environmental changes [21, 22]. In particular, radioactive contaminations after major nuclear accidents like the Chernobyl accident are characterised by a wide range of consequences on wildlife [23–25] and long environmental persistence [26]. The short-term effects of high levels of radionuclides on wild organisms are relatively well described [23]. But even if the regulation in the most contaminated places (as for example in Ukraine with the establishment of the Chernobyl Exclusion Zone greatly limiting human activities [27, 28]) promotes the presence of a large number of species (including large mammals [29]), we are still unable to describe the long-term vulnerability of populations and ecosystems [30, 31]. A good example is the bank voles (*Myodes glareolus*) living in the Chernobyl Exclusion Zone (CEZ), for which a large number of studies showed that they are still experiencing individual functional modifications along the radioactive gradient more than 30 years after the nuclear power plant accident (e.g. colouration [32], cataracts [33], organ mass [34], DNA damage response [35]). These modifications were associated with decreased populations size [36] and thus question the impact on the vulnerability of populations in contaminated places.

Here, we take the Chernobyl study case to assess the long-term consequences of such a major anthropogenic environmental change and the nature of these consequences. In particular, we attempted to conciliate the question of evolutionary processes with a more individual functionally centred study. We focused on the Eastern tree frog *Hyla orientalis*, a species of particular interest because of its relatively small dispersal radius (about 0.5 km per generation [37]) and its exposure to radionuclides in different components of the environment as it lives buried in the ground during winter, reproduces and develops in water and lives as adult in the vegetation [38]. These characteristics make this species relevant to characterise the effects of ionizing radiation (IR) and inform on the possible non-perceptible effects on other components of the biotic community, as proposed for ‘sentinel species’ [39, 40]. While over 3 years (from 2016 to 2018) the total dose rate absorbed by male tree frogs during the breeding period (ranged from 0.07 to 32.40 μSv/h) [41] was estimated to be below the no-effects threshold values (i.e. < to 42 μGy/h) determined by the International Commission on Radiological Protection (ICRP), a recent population genetics study indicated a singular evolution of CEZ populations since the accident compared to other European populations [42]. Analyses of the specific mitochondrial profile of tree frogs in the CEZ revealed a 100-fold higher rate of mitochondrial substitution and smaller effective population sizes compared to other European populations. In the same time, while several blood parameters were not shown to be dependent on total dose rate [43], the high mitochondrial substitution rate observed in the CEZ [42] pointed towards potential metabolic impairments.

The power of transcriptomic analysis, and especially the study of gene expression level, to explore the potential effects of pollutants on individual function has already been emphasised [44–46]. But transcriptomic analysis appears also particularly interesting to explore the potential effects of pollutants by assessing systemic evolutionary processes in the transcribed regions by population genetics analysis and estimate functional effects via the study of gene expression [47–49]. In this study, the de novo transcriptome assembly of *H. orientalis* allowed the comprehensive transcriptomics analysis of 87 individuals sampled in 2018 across a gradient of dose rates in the CEZ. The molecular changes potentially induced by radioactive contamination were assessed by dose–response modelling with specific emphasis on coregulation of genetic networks with environmental variables, and including the body condition index, which was used as an integrative measure of major physiological changes at the individual scale [50]. Expressed single-nucleotide polymorphisms (SNP) derived from transcriptomics data were used to analyse the population structure and genetic variation within the Chernobyl area and to assess the intensity of evolutionary processes ongoing inside the CEZ for this species.

## Results

### Negative correlation between body condition and individual dose of ionizing radiation

**Fig. 1:**
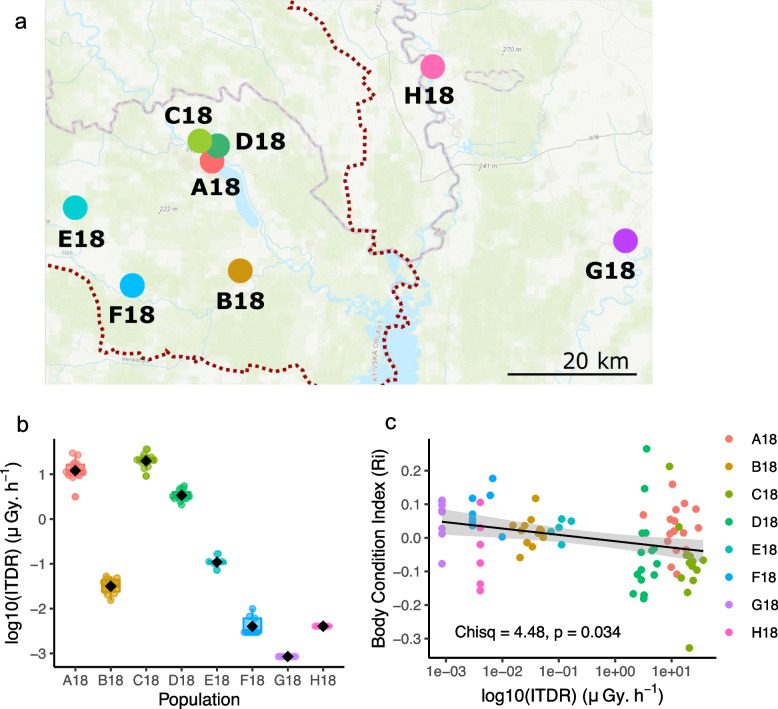
Geographical distribution, individual dose rate (ITDR) and body condition of sampled frogs. **a** Map showing the sampling sites (letters) in the Chernobyl Exclusion Zone (dashed line) and the Slavutych region. **b** Distribution of the ITDR (μGy.h^−1^) in the different sampled populations. **c** Relationship between ITDR and BCI (Ri). The linear model is represented by a black line with 95% confidence intervals

To better estimate the current exposure to IR in wild tree frogs, 87 breeding Eastern males tree frog (*Hyla orientalis*) were collected in the CEZ and in non-radiocontaminated area near Slavutych (Fig. 1a and Additional file 1: Table S1) and their individual total dose rate (ITDR) (Fig. 1b) and body condition index (BCI) were estimated. A statistically significant decrease in BCI along the contamination gradient was observed (Chisq = 4.48, *p* = 0.034), due to a higher frequency of individuals with BCI below 0 in highly contaminated sites compared to other sites (chi-square test *p* = 0.0003, 61% *n* = 43 in sites A18, C18 and D18 compared to 21% *n* = 44 in sites B18, E18, F18, G18, H18) (Fig. 1c).

### Geographically structured population and asymmetrical admixture in the CEZ

The de novo transcriptome assembly of *Hyla orientalis* was performed by using the combined set of 5.9 billion RNASeq paired end-reads from 5 tissues (tibia muscle, heart, eye, brain and testis) obtained from three specimens collected near the Slavutych locality. The E90N50 of the assembly was 1690 bp, and 95% of the conserved genes in Tetrapoda were completely assembled (BUSCO) [51]. The reference map of the *Hyla orientalis* transcriptome was defined as a set of 17,323 non-redundant assembled contigs. From the 7286 contigs encoding peptides detected by RNAseq in the muscle tissue (Tag Per Million, TPM > 0.1), 3611 (50%) corresponding proteins were also detected by proteomics (Additional file 2: Fig. S1 and Additional file 3: Supplementary Material and Methods).

**Fig. 2:**
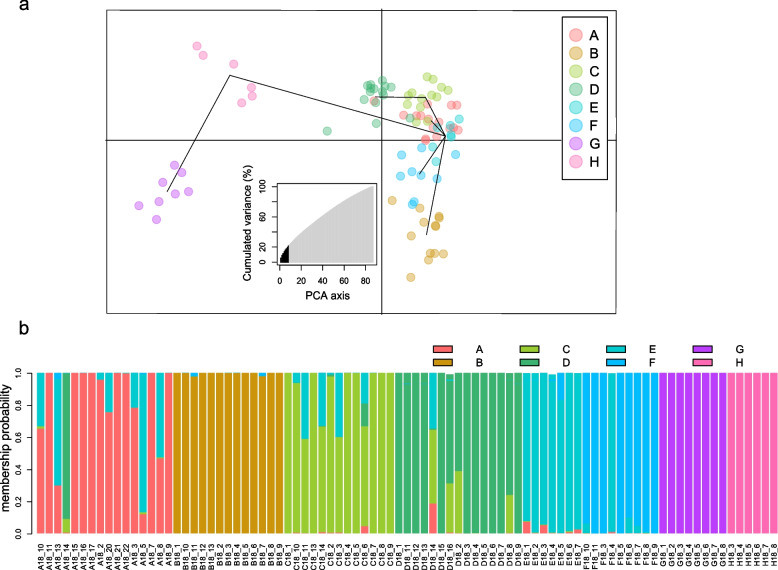
Population structure of *Hyla orientalis* in the Chernobyl region based on genetic polymorphism. **a** DAPC of 87 individuals. **b** Probability of affiliation (membership) of each individual to each sampling site

We then generated RNAseq data from tibia muscle of 87 individuals sampled in the CEZ and Slavutych region, with similar coverage (Additional file 4: Table S2, Additional file 2: Fig. S2a, and Additional file 3: Supplementary Material and Methods). The tibia muscle was dissected from one leg for all individuals as it is an abundant and easily accessible tissue in frogs, deeply innervated and with high energy demand for locomotion [52]. From these data, we collected 374,102 variants and selected 157,677 SNPs (42.15%) based on stringent quality control criteria to assess genetic diversity. The ratio of nucleotides transition/transversion (Ti/Tv) was between 2.0 and 2.2 for all individuals, as expected for this type of data [53]. To estimate the intensity of gene flow within the sampled area, and also to make an initial assessment of the relevance of the markers used, we first examined the population genetic structure. We detected that all populations inside and outside the CEZ were moderately differentiated with G’st comprised between 0.054 and 0.115. The genetic distances (G’st/(1-G’st)) were significantly and positively correlated to geographical distances, indicative of an isolation by distance (Mantel, *r* = 0.6581, *p* = 0.011). This isolation by distance remained significant even by controlling for dose rate differences (partial Mantel, *r* = 0.5915, *p* = 0.012). Thus, genetic differentiation is explained on average, by affiliation reflected by geographical distances, and not only by the averaged total dose rates (ATDR) (see Materials and Methods for more information about the ATDR). To further describe population genetic structure in the CEZ, we conducted a principal component analysis (PCA) using bi-allelic SNPs. The Slavutych populations (G18 and H18) were well separated from CEZ populations (Additional file 2: Fig. S2b), but only a relatively low variation was explained by the two first principal components (PC) (5.3% for PC1 and 3.66% for PC2). To further infer relationships between genetic clusters, we carried out a discriminant analysis of principal components (DAPC) in which populations were implemented a priori. The populations were clearly distributed according to geography, with Slavutych populations (G18 and H18) more differentiated from CEZ populations, as expected (Fig. 2a). The genetic structure of CEZ populations was also consistent with their geography. Indeed, the two southern populations B18 and F18 were close together and distant from the three central populations A18, C18 and D18, the south-west population E18 bridging these two groups. We then assessed the probability of affiliation of individuals to each sampled population based on their genotype (Fig. 2b). Individuals displayed high membership to their population defined a priori with little to moderate mixing between them, with the exception of the two Slavutych populations that were completely separated from CEZ populations. The three central populations in the CEZ, A18, C18 and D18, presented the highest number of admixed individuals, reflecting their geographical proximity. We also noted a higher proportion of admixed individuals coming from the south (E18 and F18 populations) towards the centre of the CEZ but not the converse (Fig. 2b). These observations were confirmed by non-supervised *k*-means clustering of genetic polymorphisms and by admixture analysis based on ancestry coefficient inferences, without a priori on population affiliation (Additional file 3: Fig. S2c and d).

### Functional prediction of SNP outliers correlated with individual total dose rate (ITDR)

We next aimed at describing the potential selective process ongoing in the CEZ by testing loci associated to the dose gradient and including the confounding effect due to population structure. We detected 21,432 SNPs highly correlated to ITDR (*q*-value < 0.05), scattered among expressed exons of 5283 different contigs encoding functionally characterised orthologous genes. A total of 4138 SNPs non-synonymous variants were detected and associated with genes involved in ATPase activity (GO:0016887 *q*-value < 10^−3^) and small GTPase mediated signal transduction (GO:0051056 *q*-value < 10^−2^). Finally, we found that 159 SNPs in 143 different contigs were stop-gained variants predicted to have a functional impact on orthologous proteins with function in energy metabolism including IDH3G (Uniprot P51553), ALDH2 (Uniprot P05091), and LDHA (Uniprot P00338), as well as the stress factors AHSA1 (Uniprot O95433) and EIF2AK3 (Uniprot Q9NZJ5) (Additional file 5: Table S3). The number of homozygous stop-gained mutations deviating from Hardy–Weinberg (HW) equilibrium was higher in populations highly contaminated in the CEZ compared to the others (exact test of HW equilibrium *p* < 0.01, *n* = 8 in sites A18, C18, D18, *n* = 4 in sites B18, E18, F18 and *n* = 0 for G18 and H18) (Additional file 6: Table S4).

### Negative relationship between genetic diversity indices and the averaged total dose rates (ATDR)

**Fig. 3:**
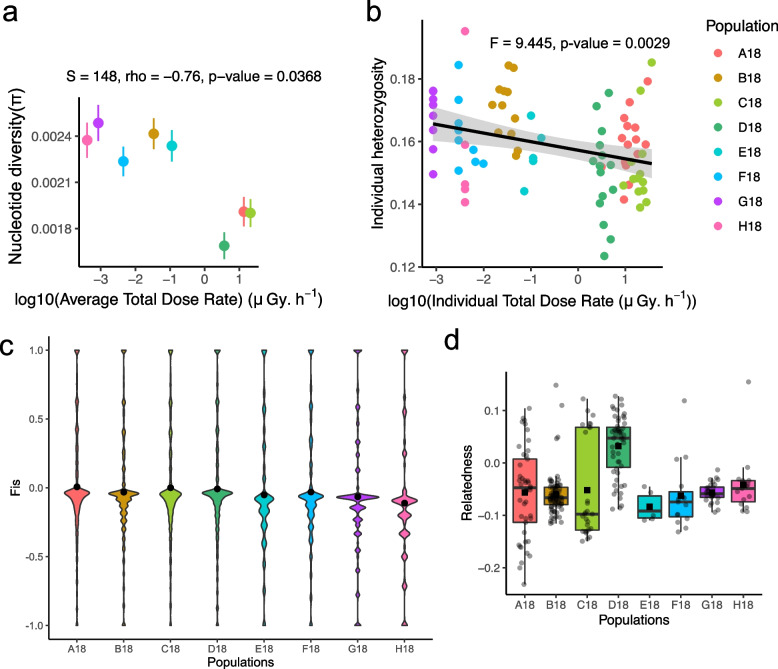
Population genetics summary and relatedness of *Hyla orientalis* in the Chernobyl region. **a** Correlation plot of nucleotide diversity (π) on averaged total dose rate (ATDR) represented at the mean and standard error per sampled population. **b** Individual heterozygosity on individual total dose rate (ITDR). The linear model is represented by a black line with 95% confidence intervals. **c** Representation of the distribution of individual heterozygosity. **d** Degree of relatedness per population

The nucleotide diversity (π) of populations located at the centre of the CEZ (A18, C18 and especially D18) was lower than the one observed at the south of the CEZ (B18, E18, F18) and in the Slavutych region (G18 and H18) (Fig. 3a). Furthermore, π and individual heterozygosity were both negatively correlated with the ATDR (respectively, *S* = 148, rho =  − 0.760, *p* = 0.037; *F* = 9.445, *p* = 0.003) (Fig. 3a, b). As decrease of heterozygosity could be a sign of increased inbreeding within the populations in the CEZ, we assessed the inbreeding coefficient (Fis) for each population. Average Fis by population was comprised between − 0.130 < Fis <  − 0.026, indicating a general excess of heterozygosity. Higher Fis indices were observed in populations highly contaminated in the CEZ (Fig. 3c) and Fis values were positively correlated to ATDR (*S* = 12, rho = 0.857, *p* = 0.011) even including only the loci at HW equilibrium (*S* = 10, rho = 0.881, *p* = 0.007). Thus, the populations studied here do not show general sign of inbreeding, despite a decrease in genetic diversity in most contaminated populations. We next assessed variability of relatedness inferred by pairwise kinship coefficient between sites. Higher variations in relatedness were observed within highly contaminated populations in the CEZ (Fig. 3d) (*z* = 5.73, tau = 0.253, *p* = 9.93.10^−9^) with notably a higher percentage of individuals with relatedness higher than zero in the three most exposed sites (15%) than in lower exposed sites (2%) (Chisq = 41.26, *p* = 1.33.10^−10^). Together, these results indicate that individuals sampled in highly contaminated area in the CEZ do not show sign of higher inbreeding but display higher degree of relatedness.

### Dose dependent effects in the CEZ assessed by transcriptomics clustering analysis

Individuals were discriminated in two main groups by hierarchical clustering of contig expression (Additional file 2: Fig. S3). To test how ITDR contributes to this observed pattern, we tested the correlation between individual cophenetic distances (as an estimate of similarity between individual) and ITDR. We found a significant positive correlation between cophenetic distances and ITDR (Mantel, *r* = 0.121, *p* = 1.10^−4^). Importantly, this correlation remained significant even by controlling for genetic distances (partial Mantel, *r* = 0.126, *p* = 1.10^−4^) or geographical distances (partial Mantel, *r* = 0.101, *p* = 2.10^−4^). Thus, these results highlight that changes in expression patterns observed by transcriptomics analysis are correlated to the ITDR, and importantly this correlation does not depend only on geography or genetics.

### Impact of long-term exposure to radiocontaminants in the CEZ by transcriptomic analysis

The differential expression analysis of 17,323 contigs was performed using individuals sampled outside the CEZ as reference. Comparison of fold changes was correlated (rho > 0.63) using either reference populations (G18 or H18) or a lowly contaminated site (F18) showing that the dose effects were prominent, and choice of reference sites did not impair the identification of differentially expressed contigs (Additional file 2: Fig. S4). Using the most distant site (G18) as reference, we found 477 differentially expressed genes (DEG) in common in the pair-wise comparative analysis made for the highly contaminated sites (A18, C18, D18) and 367 DEG common for the lowly contaminated sites (B18, E18, F18). The differentially expressed contigs were involved in pathways involved in oxidoreductase activity (GO:0016705, *q*-value < 1.10^−2^) and glycolysis and gluconeogenesis (hsa00010, *q*-value < 1.10^−2^) (Additional file 7: Table S5).

**Fig. 4:**
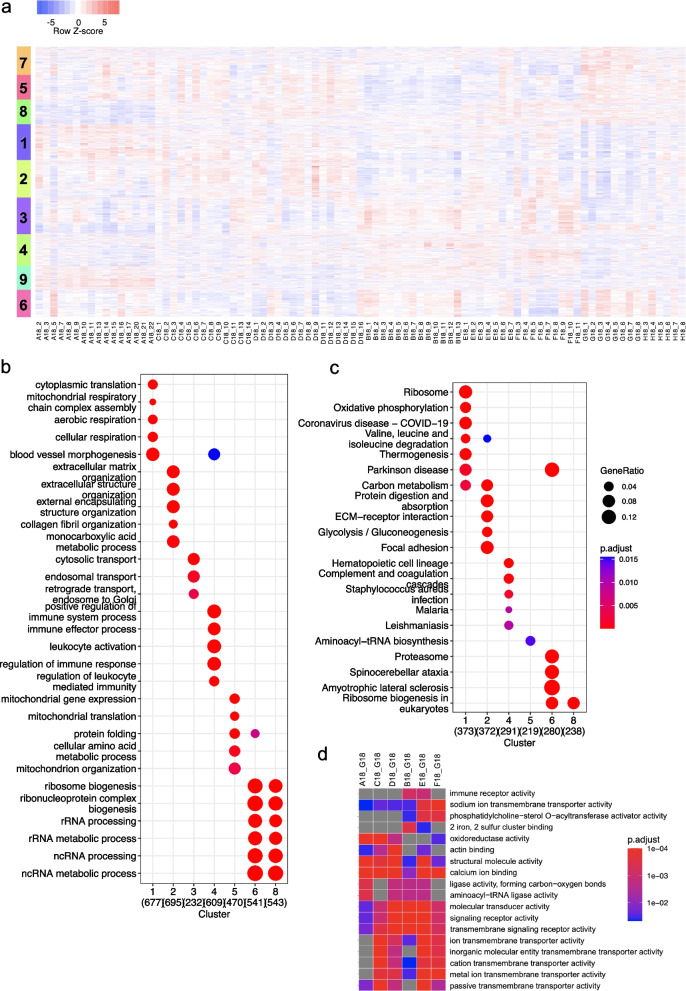
Analysis of gene expression by transcriptomics analysis in the 87 individuals. **a** Unsupervised hierarchical clustering of normalised expression of 5735 contigs differentially expressed in at least one population (using G18 individuals as control). Top dendrogram corresponds to the clustering of individuals based on overall contig expression, highlighted as two main clusters (black and blue). The expression levels of contigs (as Row *Z*-score) are represented as follows: low expression (blue), moderate expression (white) and high expression (red). **b** Enrichments of Gene Ontology Biological Process and **c** KEGG in the 9 different clusters obtained by hierarchical clustering. The numbers in brackets correspond to the total number of contigs in each cluster (bottom, *x*-axis). **d** Treeplot based on semantic similarity of GO molecular function obtained after by gene set enrichment analysis (GSEA). The different biological pathways enriched in each gene set (comparative analysis between each site and using G18 as a reference) are indicated at the bottom of the heatmap. The colour-code from blue to red depicts the *q*-value of the enriched GO terms (grey colour indicates no enrichment)

We then analysed the transcriptomics changes potentially associated with the environmental changes in the 87 individuals. Several groups of contigs were characterised by relative higher expression in individuals sampled mainly from sites A18, C18 and D18 (clusters 1 and 2), while a second group of contigs was expressed at low levels in individuals from sampling sites A18, C18 and D18 (cluster 3,4 and 6) (Fig. 4a). These results show that individuals sampled in highly contaminated sites inside the CEZ have a specific transcriptome signature that discriminates them from individuals originating from lowly contaminated sites in the CEZ or outside the CEZ. We then assessed which biological pathways were deregulated in individuals sampled in the CEZ by analysing the GO biological processes, GO molecular functions and KEGG pathways enriched in the different clusters obtained after hierarchical clustering. Contigs present in the cluster characterised by higher levels of expression in the highly contaminated sites were enriched (among others) in aerobic respiration (GO:0009060, *q*-value < 10^−6^), glycolysis and gluconeogenesis (hsa00010, *q*-value < 10^−5^) and muscle cell development (GO:0055001, q-value = 0.0016). Contigs lowly expressed in highly contaminated individuals in the CEZ were involved in immune response (GO:0002252, *q*-value < 10^−8^) and tumor necrosis factor signalling (GO:0032640, *q*-value < 10^−2^) (Fig. 4b, c). Importantly, adjusting contigs expression by the genetic distances led to clusters of contig expression with similar GO term composition (Additional file 2: Fig. S5). A complete list of the biological pathways enriched in each cluster is provided in Additional file 8: Table S6. Gene set enrichment analysis was then performed on the different sets of differentially expressed genes (using G18 as a reference, as before). The differentially expressed genes from highly contaminated sites (A18, C18 and D18) were enriched in proteins involved in oxidoreduction processes, while differentially expressed genes in the lowly contaminated sites (B18, E18 and F18) were enriched in proteins with function in lipid metabolism (phosphatidylcholine − sterol O − acyltransferase activator activity). Signalling transducer molecules and structural components of actin cytoskeleton were enriched in all gene sets (Fig. 4d).

**Fig. 5:**
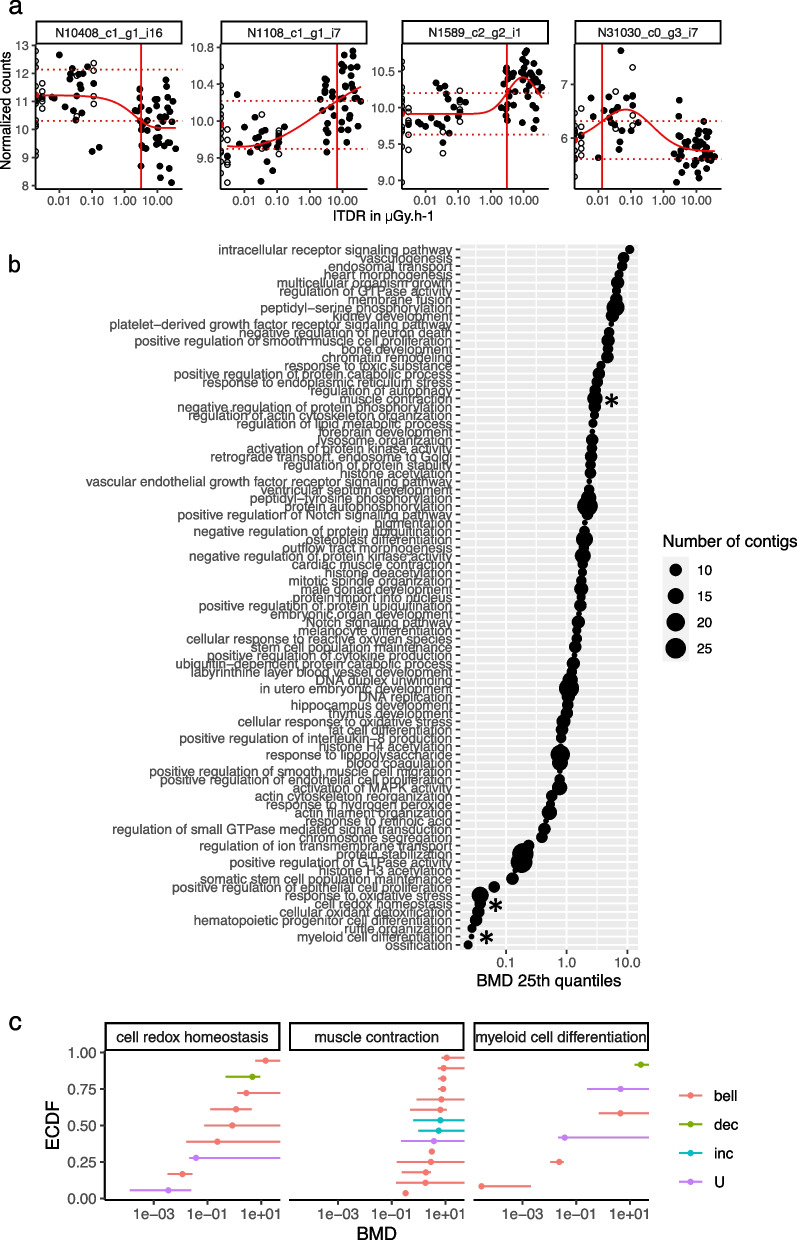
Dose–response modelling and derivation of benchmark mark dose (BMD_1SD_) from the transcriptomics data. **a** Examples of dose–response modelling for several contigs showing the expression values (black dots) along the dose gradient. The dose–response model computed (red line) is used to determine the BMD_1SD_ (vertical line), defined as the minimal dose for which the signal reach the benchmark response (BMR = y0 ± SD) with the modelled value at null dose and the SD the estimated residual standard deviation around the model (dotted horizontal lines). The ITDR (in μGy.h^−1^) as a log scale on the *x*-axis, responses at the control sites (G18 and H18) appear as half circles on the *y*-axis. **b** Distribution of the enriched GO biological process summarised by the 25th percentile of the BMD_1SD_ values from all modelled contigs belonging to the considered GO term. The size of each point codes for the number of modelled contigs in each GO term. Asterisk symbol (*) indicates the 3 GO term for which empirical cumulative distribution function (ECDF) is shown as example in **c**. **c** Example of ECDF plots used to estimate BMD_1SD_ shown in **b** (*). The 95% confidence intervals are indicated for each selected contigs categorised in 3 different GO biological processes. The dose (in μGy.h.^−1^) is indicated on the *x*-axis. The trend of the dose–response curves fitted for each contig is indicated by the colour code (bell: bell-shape, U: U-shape, dec: decreasing (downregulated), inc: increasing (upregulated)

We then modelled contigs expression profiles along the dose-gradient [54] and computed benchmark dose (BMD_1SD_) to rank the sensitivity of the deregulated GO pathways (Fig. 5a). The subset of contigs with dose–response models were notably involved in the response to oxidative stress (GO:0006979, *q*-value = 0.016), cellular response to lipid (GO:0071396, *q*-value = 0.012) and muscle system process (GO:0003012, *q*-value = 0.042), thereby showing a deregulation of contigs involved in these biological pathways in function of the ITDR. The complete list of the deregulated pathways is provided in Additional file 9: Table S7. Ranking of the BMD_1SD_ highlighted that the pathway involved in reduction of oxidative stress was deregulated at lower ITDR compared to contigs related to muscle contraction (BMD_1SD_ < 10^−2^ μGy.h^−1^ and about 1 μGy.h^−1^ respectively) (Fig. 5b, c).

**Fig. 6:**
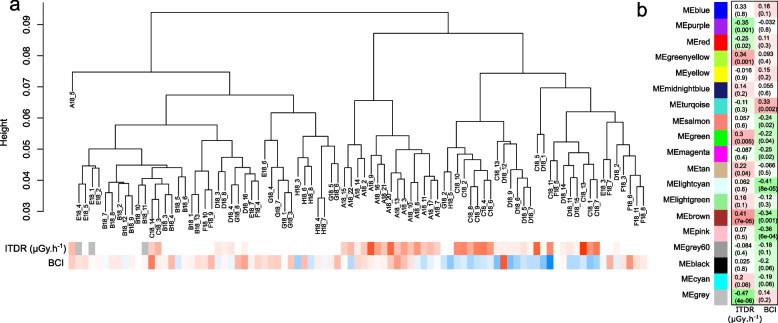
Association between contig expression and individual variables (BCI and ITDR) by weighted gene co-expression network analysis (WGCNA). **a** Dendrogram of samples based on their Euclidean distance and association with ITDR (μGy.h^−1^) and BCI (Ri). The colour codes for the values of the ITDR or BCI: low (blue), moderate (white) and high (red) values. Grey colour indicates missing values. **b** Traits associations with module eigengene correlated to the BCI or ITDR

We then applied a non-supervised machine learning methods based on weighted gene co-expression network analysis [55] to identify modules of co-expressed contigs associated with variations of the BCI and ITDR (Fig. 6a). One module was significantly associated with ITDR and enriched in contigs with function in mitochondrial ATP synthesis coupled electron transport (*q*-value < 10^−19^) and aerobic respiration (*q*-value < 10^−27^). One other module was associated with the body condition index (BCI) and contained contigs involved in extracellular matrix structural constituent (*q*-value < 10^−39^) (Fig. 6b and Additional file 10: Table S8). The correlation between the module eigengene and ITDR remained significant after controlling for genetic distances (*p*-value = 0.0017, see Additional file 3: Supplementary Material and Methods). Finally, modules correlated to ITDR or BIC after correcting contigs expression by genetic distance were enriched in ATP synthesis (*q*-value < 10^−4^) and oxidative phosphorylation (*q*-value < 10^−4^) (Additional file 2: Fig. S6 and Additional file 11: Table S9). Together, these results demonstrate that the transcriptional regulation of genes involved in several biological processes like ATPase activity, hexose metabolism and muscle function are affected by IR, and these changes are correlated to a decreased BCI.

## Discussion

Tree frog (*Hyla orientalis*) populations living in highly contaminated sites inside the CEZ showed lower nuclear genetic diversity and higher relatedness, as a result of small population sizes. In addition to the underlying genetic drift process, we highlighted stop-gained mutations and changes in transcriptional profile strongly associated with the individual dose received which are predicted to have or support functional consequences in genes involved in energetic metabolism, and in turn could explain phenotypic changes including body condition index.

The analysis of genetic variants showed a significant negative correlation of average nucleotide diversity and average individual heterozygosity with ITDR indicative of genetic drift inside the CEZ, likely due to small effective population sizes. Decreased population sizes associated with the contamination of the environment is also corroborated by the higher propensity of sampled individuals to be more related in the most contaminated sites. Our data on inbreeding coefficient did not reveal signs of higher consanguinity inside the CEZ at the level of the genome but rather high variability in relatedness with notably higher degree of relatedness in the most contaminated sites.

By analysing genetic population structure, we highlighted that populations inside and outside the CEZ were genetically differentiated. The Slavutych populations were more differentiated compared to the ones collected inside the CEZ, pointing towards genetic specificity of the CEZ populations even if the geographical distance between Slavutych and CEZ populations is low (36 km for the closest and 66 km on average). The presence of higher number of admixed individuals in the centre of the CEZ indicates a directed gene flow towards the most contaminated places which can reflect past or ongoing migrations from low towards higher contaminated sites, either as a consequence of recolonisation of declined frog populations or the decrease of anthropogenic activities in the centre of the CEZ, as proposed for barn swallows [56] and bank voles [57]. The fact that population size is smaller in the most contaminated site support the notion that this dynamic is fuelled by a higher mortality or lower reproduction [58]. Thus, migrations and elevated mutations seem not sufficient to offset the genetic diversity loss by bringing new genetic variants in the most contaminated sites of the CEZ.

While we cannot exclude the fact that lower genetic diversity observed in the most contaminated places could be the result of past strong population bottlenecks at the time of the accident (corroborated by the massive functional impacts on amphibians observed at the time of the accident) [23], previous simulations carried out on a mitochondrial marker suggest current small effective population sizes [42]. Current small effective population sizes can be indicative of decreased individual viability in the most contaminated places. Using BCI as an integrative trait to evaluate individual performances linked with energetic status [50], and sometimes proposed as a metric of fitness [59, 60], we showed a negative correlation of this parameter with ITDR. Thus, the exposure to IR is associated with functional modifications in the CEZ. Because of the wide range of mechanisms implicated in BCI variations [61], non-supervised analysis of individual expression variations was performed in order to identify the main functional pathways associated with exposure to IR and mass decrease. The transcriptomic analysis pointed towards an effect of exposure to IR inside the CEZ, as low versus high contaminated sites were discriminated based on gene expression. The gene expression pattern (observed at the individual level) is explained by ITDR, and this relation remained significant by controlling for geographical distance or genetic distances. The changes in gene expression observed in the CEZ are thus not solely a consequence of genetic differentiation inside the CEZ but rather the result of ITDR differences, despite other environmental characteristics specific to the CEZ cannot be completely excluded. Furthermore, the biological effects detected by the transcriptomic analysis, collectively point toward deregulations of mitochondrial activity and muscle functions in frogs living in the CEZ, which fits with the known effects of chronic exposures to IR [62–65].

The results of the gene expression analysis suggest a substantial role of radioactive contamination on functional differences between individuals, impacting notably major energetic pathways. The proximate mechanism at the origin of these modifications remains unknown. Indeed, it could be both the effect of plastic changes and an adaptive response on a trait selected since several generations, as gene expression levels are known to be heritable and thus potentially under selection [66, 67]. Genetic drift induced a lower range of transmitted variations, which can have functional consequences on which selection can act and thus render the plasticity particularly valuable if the functional changes bring individual closer to a fitness optimum [20]. Moreover, the functional response observed at the transcriptomic level appears constrained (as individuals from the most contaminated places show very similar gene expression patterns) and may thus be indicative of an adaptive response. A more in-depth analysis of expression diversity in each site for the different contigs could be a way to evaluate the nature of this response and which functions are involved [68, 69]. Although this response seems non-optimal, as it does not compensate the effective population size decline inferred in the most contaminated places, this does not exclude some of these changes from being subject to selection.

Using RNaseq data to analyse SNP represents a promising and cost-effective alternative compared to whole genome sequencing, especially for non-model species for which no reference genome is available. The main limit, however, is that SNP are obtained only from exons that must be expressed at sufficient levels. If we cannot exclude that detection of SNP from gene expression might lead to bias in estimating genetic parameters, recent analysis shows that RNA-seq data can be used with good confidence to detect SNPs [70, 71]. It is not yet possible to discriminate genetic variants only correlated with radiocontamination from loci genuinely under selective process, but many genes involved in response stress and ATPase activity and correlated with ITDR may thus be indicative of selective process on these functions. Several loci strongly associated to ITDR were found to be stop-gained mutations that are predicted to affect the function of several proteins involved in mitochondrial energy production (IDH3G, ALDH2, LDHA). Noteworthy, we observed an increased number of individuals with stop-gained mutations in these genes in the CEZ. Because of the predicted functional deleterious impact of these changes, it is most likely that these genetic variants do not represent cases of selection but rather de novo radiation-induced mutations that accumulate in the small population of frogs through inbreeding. Ultimately, these changes are in line with the transcriptomics data that showed changes in ATP metabolic process and glucose metabolism. These changes in metabolism observed at the molecular level in individuals exposed to higher IR doses were correlated to a decreased BCI. In addition, other biological processes were detected by the transcriptomic analysis, including change of expression of genes involved in muscle function and unfolded-protein stress response. As chronic exposure to relative low levels of IR was shown to alter the structure of myofibrils [64], it is possible that our results highlight a disruption of myofibers in frog muscles, an organ with high energy demand and rich in mitochondria. Together, these data collectively point toward a frequent impairment of mitochondrial function, which could cause the degraded body condition of individuals living in the CEZ. Despite congruent functional changes in individuals found in highly contaminated areas of the CEZ, our data do not demonstrate the effects of a selection of traits favourable to maintain frog population in the CEZ. Rather, the most parsimonious hypothesis is that frog populations inside the CEZ are maintained, in part, by migration from other less contaminated territories.

More than 30 years after the Chernobyl nuclear power plant accident, numerous studies showed contemporary functional modifications in wildlife associated with differences in exposure to IR [35, 72–75]. While generalisation to every exposed species is not possible [31, 76, 77], and the consequences at the ecosystem scale and the mechanisms involved in these changes remain subjected to debate [31], we highlighted the interest of taking an evolutionary perspective to interpret gene expression differences. The functional changes observed in the most contaminated places are expected to influence fitness and thus show the need to study the long-term specific processes that are taking place on wildlife in polluted areas. Mass loss and body condition in particular are expected to influence reproduction by modifying reproduction investment [78, 79]. These fitness costs and the transmission of functional changes through generations should be better addressed in the future, for example with the establishment of field-based reciprocal transplants [80]. We also highlighted the potential key role of energy metabolism in response with radiation exposure from a body of evidence: changes in body condition, differential expression of genes involved in energy metabolism and association of dose rates with genetic variants on genes involved in mitochondria energy production. More generally, we analysed a specific type of response to persistent environmental change which combines an effective small population size coupled to an increase of deleterious mutations in some loci that directed functional plastic changes and viability loss. While these changes might allow the persistence of populations facing the radiocontaminated environment, this study questions the constraints that an eroded genetic diversity places on possible adaptation, and in particular of a ‘genetic assimilation’ which could lead to the fixation of initially plastic phenotypes [81, 82].

## Conclusions

The combination of gene expression study, SNP data and population genetic analyses allows us to identify genetic disturbances that were previously inaccessible [42]. In a radio-contaminated environment such as the CEZ, even several decades after the nuclear accident and with total absorbed dose rates that the ICRP has so far estimated to have no deleterious effects on frogs [41, 83], individuals inhabiting the CEZ still display signs of significant disturbances such as genetic erosion, disruption of transcriptomic/metabolic pathways and changes in physiological traits. These changes are consistent with altered metabolism and fitness costs resulting from long-term radiation exposure, but the causal relationship with radiation remain to be established. Future studies in radio-contaminated areas should use a combination of “omics” data and population genetics analyses to investigate rigorously possible hidden deleterious effects.

## Methods

### Sampling of Hyla orientalis and dosimetry

The individuals used in the present study correspond to sampling made in Ukraine in 2018, as described before [42, 43]. Briefly, *Hyla orientalis* calling male individuals were collected during the night in May–June 2018 from 8 different wetlands in Ukraine, including 6 sites in the CEZ (A18, B18, C18, D18, E18, F18) and two sites 10 km and 45 km east from the Chernobyl NPP (G18 and H18, respectively). Thereafter, these sites are referred to as populations in the sense of “population sample.” The 6 sites inside the CEZ covered a gradient of ambient dose rates ranging from 0.1 to 16.2 μGy.h^−1^, while dose rates close to background were measured for the 2 sites outside the CEZ (0.04 μGy.h^−1^) (radiometer MKS-AT6130, Atomtex). Absence of trace metallic contamination (including uranium, thorium and plutonium isotopes) in the sites G18 and H18 was confirmed by elemental analysis of water samples (see Elemental Analysis). Individuals were sampled inside the CEZ (*n* = 73) and in the 2 non-contaminated sites (G18 and H18) (*n* = 14). Three additional individuals were sampled to produce the reference transcriptome (see Additional Material and Methods). After capture, frogs were kept individually in a wet plastic container overnight, measured, weighted and dissected as described before [42, 43]. The tibia (*gastrocnemius*) muscle was dissected from one leg for all individuals as it is an abundant and easily accessible tissue in frogs, deeply innervated and with high energy demand for locomotion. The other leg was used for reconstruction of the internal contamination (see below). All tibia samples used for RNA extraction were immerged in individual tubes with 1 ml of RNA later (Sigma) and kept in liquid nitrogen. Internal contaminations of ^90^Sr and ^137^Cs were measured for each individual in bone and leg muscles, respectively, in the International Radioecology Laboratory (IRL) (Slavutych, Ukraine) to reconstruct the total internal dose, as described before [41, 42]. The individual total dose rate (ITDR) in μGy.h^−1^ was reconstructed from both internal and external exposures using EDEN [84], considering both soil and water activities as before. Averaged total dose rates (ATDR) were obtained by computing the mean ITDR per site. All samples were deep frozen until their transport to the Institute for Radiological Protection and Nuclear Safety (IRSN) laboratory (Cadarache, France).

### Elemental analysis on water samples

Water samples collected on reference sites (G18 and H18) were acidified to 2% (v/v) HNO3 with Ultra-Pure JT Baker, 70% (v/v) nitric acid, and analysed for trace metals and major elements with ICP-MS (7800 Agilent, Tokyo, Japan) and ICP-AES (Optima 8300, Perkin Elmer, Waltham, Massachusetts, USA) respectively, according to already published procedures [85] (Additional file 12: Table S10).

### RNA extraction and sequencing libraries

The 87 individuals sampled in and outside the CEZ were subjected to RNA-Seq analysis. Total RNA was extracted from tibia muscle with the Quick-DNA/RNA Miniprep kit (Zymoresearch) following manufacturer’s instructions. Integrity and RNA concentrations were assessed on RNA Nano Chips (Bioanalyzer 2100, Agilent). All samples had an RNA integrity index > 8 and were used for library preparation using the Stranded mRNA Prep Ligation kit with ITD UD index (Illumina) following instructions. After quality check (size and concentration) on DNA1000 Chips (Bioanalyzer 2100, Agilent), libraries were sequenced on a NovaSeq6000 platform to produced 100 or 150 bases long paired-end reads using S4 flow cells (Clinical Research Sequencing Platform, Broad Institute, MIT, United States). All fastq files were quality checked with an in-house pipeline relying on fastqc and fastx-toolkit.

### Transcriptomics analysis

The de novo assembly of *Hyla orientalis* transcriptome was made with Trinity [86] using the complete dataset obtained from five different tissue type (tibia muscle, heart, eye, brain and testis) obtained from 3 additional individuals sampled in non-contaminated site. Completeness of *Hyla orientalis* transcriptome assembly was assessed by BUSCO [51] analysis. We searched similarities to SwissProt [87] and UniRef90 [88] (E < 1e^−3^) using blastx and blastp [89] with the assembled contigs and predicted ORF respectively. All annotations were collected in a mySQLite relational database using *Trinotate* [90] for annotating putative functional characteristics. The quality of the assembled contigs was confirmed by comparing the sequence of the predicted ploypeptides to proteomics data obtained by Mass spectrometry from 3 muscles of the same individuals. Proteins were extracted as before [64]. Peptides were analysed using liquid nanochromatography online in front of an Orbitrap Fusion Lumos Tribrid mass spectrometer (Thermo Fisher Scientific) as previously describe [91]. Transcript abundance estimation were mapped against the de novo assembled *Hyla orientalis* transcriptome using Bowtie2 [92] and quantified by RSEM [93]. Differential analysis was made by Deseq2 [94]. Dose–response modelling and BMR (benchmark response) estimation were computed with DRomics [54]. Weighted gene co-expression network analysis was performed with WGCNA [55]. See Additional file 3: Supplementary Material and Methods and Additional file 2: Fig. S1 for details on methods for the transcriptomics data analysis.

### Variant detection

Uniquely mapped reads were processed for variant calling as described in GATK’s best practices for RNA-Seq data [95, 96]. Briefly, mate information was fixed, duplicates marked and reads splited with the GATK function SplitNCigarReads. No base recalibration and base quality reassignment could be performed as prior SNPs information are missing for this species. Germline variants were detected in each sample using GATK HaplotypeCaller 4.2.0.0 with the options -native-pair-hmm-threads 46 –pcr-indel-model NONE -ERC GVCF. Individual vcf files were then used for joint genotyping with the GATK’s functions GenomicsDBImport and GenotypeGVCFs to produce a single gvcf file. The following filtering steps were then performed: (i) vcftools [97] was used with the options –remove-indels –max-missing 0.75 –recode to remove sites were at most 25% of the genotypes were missing, (ii) we then applied the following filters recommended by GATK’s best practices for RNA-Seq SNPs calling: -filter “QD < 2.0” –filter-name “QD2” -filter “QUAL < 30.0” –filter-name “QUAL30” -filter “SOR > 3.0” –filter-name “SOR3” -filter “FS > 60.0” –filter-name “FS60” -filter “FS > 30.0” –filter-name “FS30” -filter “MQ < 40.0” –filter-name “MQ40” -filter “MQRankSum < -12.5” –filter-name “MQRankSum-12.5” -filter “ReadPosRankSum < -8.0” –filter-name “ReadPosRankSum-8”.

### Population genetics summary statistics

Nucleotide diversity (π) for each population is the mean of nucleotide diversity for each contig calculated as the average number of nucleotide differences (θπ) divided by the total number of positions (segregating or not segregating sites) except positions with missing data in any individual. The individual heterozygosity ratio was calculated as the number of heterozygous positions on the number of segregating sites used for an individual. π and the individual heterozygosity were computed with DnaSP [98]. Expected (He) and observed (Ho) Heterozygosity of each population for each contig were computed with PLINK [99] and inbreeding coefficient (Fis) as 1—(Ho/He) [100]. We then analysed the distribution of Fis values for the different loci for each population. To evaluate the contribution of loci with extreme values, we compared Fis between populations solely for loci at the Hardy–Weinberg equilibrium.

### Population structure

Genetic variability was computed by pairwise Hedrick G’st [101] analysis between all populations with vcfR v.1.12.0 [102]. Multivariate analyses were performed among bi-allelic SNPs to take into account the different loci. Principal component analysis (PCA) and discriminant analysis of principal component (DAPC) were conducted with the adegenet v.2.1.3 package [103]. For DAPC, eight principal components were retained representing 21% of the total variance to keep a sufficient power of discrimination between observed and random effects and to avoid over-fitting (a-score criterion). We retained and interpreted the two first discriminant functions which were the most informative. Group memberships of DAPC were represented with a “STRUCTURE-like” barplot to investigate differentiation between populations and their level of admixture. In addition, ancestry estimation was obtained from the individual SNP data with the algorithm *admixture* using *k* = 7 [104]. In order to make inferences about the potential effective size differences between populations, we estimated individual pairwise kinship coefficient [105] for individuals which are affiliated to more than 95% to their initial population (to get rid of potentially recent migrants). The identification of genetic clusters without a priori was made with k-means clustering with all PCs and a number of cluster *k* = 8, corresponding to the eight populations sampled as a maximum of cluster.

### Identification of loci potentially under selection

To identify loci potentially under selection along the contamination gradient, we used a Latent Factor Mixed Model (LFMM) implemented in LEA v.2.8.0 package [106]. This model provides a genome-wide ecological association study, by fitting a statistical model including population structure, which permits to test the association between genetic polymorphism and an environmental variable. Here, by running first non-negative matrix factorisation algorithms (sNMF), 3 clusters were selected to optimise the cross-entropy criterion. LFMM were then performed with 5 runs (iterations = 6000, burnin = 3000) with the ITDR as environmental variable and outliers identified using *q*-values (FDR, Benjamini-Hochberg) < 0.05. Genomic variant annotations and functional effect prediction were made with SnpEff [107]. Biallelic SNP outliers predicted to cause homozygous stop-gained mutations were tested for deviating from the HW equilibrium using the R package HardyWeinberg version 1.4.1 using the HWExact test option.

### Statistical analysis of the link between ionizing radiation and body condition, genetic diversity, and expression

Body condition of Eastern tree frogs was estimated with the residuals of the regression of log10(body mass) against log10(snout-vent length) (Ri) [108, 109]. Linear mixed models were used to test the link between log(ITDR) and body condition index with site as random factor. Linear models were used to test the link between log(ITDR) and individual heterozygosity. Correlations between genetic diversity indices (π and Fis) and ATDR were tested with non-parametric Spearman’s rank tests. To investigate potential changes in variance in relatedness along the contamination gradient, correlation between ATDR and the residuals of relatedness for each site, calculated as the absolute value of relatedness from median of relatedness of each site, was tested with non-parametric Kendall’s test. At the population level, correlation between log(geographical distances) (in meter, m) and genetic distances (G’st/(1-G’st)) were tested using Mantel’s test [110]. The dependence of this correlation on pairwise ATDR absolute difference was tested using partial-Mantel test. At the individual level, Mantel tests were used to test the correlation between cophenetic distances (i.e. interindividual similarity) obtained by the hierarchical clustering of 5735 differentially expressed contig and log(ITDR) or log(geographical distances) (see also Additional Material and Methods). The dependence of these correlations on log(geographical distances) and genetic distances respectively was tested with partial Mantel tests. Mantel and partial-Mantel tests were carried out on *vegan* v.2.5–6 package [111] and significance estimated by permutation’s test using 9999 permutations. Threshold of significance were all set to *p* < 0.05. All tests were carried out on R version 3.6.1 (A language and environment for statistical computing. R Foundation for Statistical Computing, Vienna, Austria. URL https://www.R-project.org/).

## Supplementary Information

**Additional file 1: Table S1.** Sampling sites, doses and individual parameters measured in 87 sampled frogs including weight, length, width, BCI (Ri), Individual Total Dose Rate (ITDR) and Average Total Dose Rate (ATDR).

**Additional file 2: Fig. S1.** Experimental design of the transcriptomic analysis on *Hyla orientalis*. **Fig. S2.** Contigs expression and population structure analysis. a. PCA on RNAseq expression data. b. DAPC on genetic variants obtained from the 87 individuals. c. Results of the K-mean clustering of individuals based on genetic polymorphisms. d. Admixture analysis made on the SNP from the 87 individuals to assess ancestry coefficient. **Fig. S3.** Bootstrap analysis after hierarchical clustering of 5,735 contigs expressed in 87 individuals. AU (Approximately Unbiased) p-value and BP (Bootstrap Probability) value are represented at each node in red and green respectively. AU *p*-value (computed by multiscale bootstrap resampling) is a good approximation of unbiased *p*-value. Branches with confidence intervals > 90% are highlighted in red. **Fig. S4.** Comparison of the differential expression analysis (*n* = 17,323 contigs) using G18, H18 or F18 populations as references. The correlation of the FC obtained by using the different reference sites is indicated in each comparative analysis. The Pearson correlation coefficient *rho* is indicated at the top. **Fig. S5.** a. Unsupervised hierarchical clustering of normalised expression of 5,735 contigs differentially expressed in at least one population (using G18 individuals as control) after removing the effects of genetic distances as covariables. The expression levels of contigs (as Row Z-score) are represented as follows: low expression (blue), moderate expression (white) and high expression (red). b. Enrichments of Gene Ontology Biological Process in the different clusters obtained by hierarchical clustering. The numbers in brackets correspond to the total number of contigs in each cluster (bottom, x-axis). **Fig. S6.** Association between contig expression and individual variables (BCI and ITDR) by WGCNA after adjusting the expression for genetic distances and removing low abundance genes (*n* = 13,641). Traits associations with module eigengene correlated to the BCI and ITDR are indicated for each module (correlation coefficient and *p*-value are indicated).

**Additional file 3. **Supplementary material and methods.

**Additional file 4: Table S2.** NGS quality control of RNAseq 87 individuals, including RNA index Number (RIN), read coverage and read quality (Phred score as Q30).

**Additional file 5: Table S3.** Summary of SNPs correlated with ITDR in the CEZ. The result of tblastx giving the closest human Uniprot orthologue is indicated, as well as the results from SNP calling with GATK, variant prediction from SnpEff and the DEG obtained by DESeq2.

**Additional file 6: Table S4.** a. List of the 159 SNPs in the 143 different contigs presenting a stop gained variants for the three level of contamination: Red (populations in the centre of the CEZ exposed to “higher” dose rate: A,C,D), Yellow (“medium” dose rate for populations BEF), Green (“lower” dose rates, for populations G,H). We indicate the *p*-value of the Fisher exact test for hardy–Weinberg equilibrium: green circle not significant, yellow circle significant with p-value ranging from 0.01 to 0.05, red circle significant with *p*-value < 0.01. b. Number of significant Fisher exact test p-value for the three different radioactive contamination area (high = red; yellow = medium; green = low). c. Genotype distribution (Wt = wild type; + stop gained mutation) for the different genes presenting a *p*-value < 0.01 and for the three different radioactive contamination area.

**Additional file 7: Table S5.** Functional enrichement of differentially expressed contigs in common after categorisation of population living in highly contaminated sites (named as cluster 1: A18, C18, D18 versus G18) and low contaminated sites (named as cluster 2: B18, E18, F18 versus G18). a. KEGG, b. Molecular Function, c. Biological Process.

**Additional file 8: Table S6.** Functional enrichment in each of the 9 clusters obtained by hierarchical clustering of differentially expressed contigs (*n* = 5,735). a. KEGG. b. Biological process. c: Molecular Function.

**Additional file 9: Table S7.** Enrichment of GO Biological Process after dose–response modelling of contig expression by Dromics.

**Additional file 10: Table S8.** Enrichment of GO Biological Process and GO Molecular functions in module eigengenes obtained from weighted gene co-expression network analysis.

**Additional file 11: Table S9.** Enrichment of GO Biological Process functions in module eigengenes obtained from weighted gene co-expression network analysis using the matrix of expression filtered for lowly expressed genes and corrected by genetic distances.

**Additional file 12: Table S10.** Concentration of heavy metals and radionuclides measured on the two reference sites near Slavutych (G18 and H18). Recommended guidelines are indicated for each element (when existing). Na: Not available.

## Data Availability

All data generated or analysed during this study are included in this published article, its supplementary information files and publicly available repositories. RNASeq data are available under the Gene Expression Omnibus (GEO) accession number GSE211060 at the address: https://www.ncbi.nlm.nih.gov/geo/query/acc.cgi?acc=GSE211060. [112].

## Abbreviations

ATDR: Average total dose rate
BCI: Body condition index
CEZ: Chernobyl Exclusion Zone
DAPC: Discriminant analysis of principal component
FDR: False discovery rate
Fis: Inbreeding coefficient
Gst: Coefficient of genetic differentiation
HW: Hardy-Weinberg
ICRP: International Commission on Radiological Protection
ITDR: Individual total dose rate
IR: Ionizing radiation
LFMM: Latent Factor Mixed Model
PCA: Principal component analysis
RNASeq: RNA high-throughput sequencing
RNA: Ribonucleic acid
SNP: Single-nucleotide polymorphism
WGCNA: Weighted correlation network analysis
π: Nucleotide diversity
