# Supplementary material for: Population transcriptogenomics highlights impaired metabolism and small population sizes in tree frogs living in the Chernobyl Exclusion Zone
Source: BMC Biol. 2023 Jul 31;21:164. doi: 10.1186/s12915-023-01659-2 (PMC10391870; doi:10.1186/s12915-023-01659-2)
Supplement: Supplementary file 2 — Additional file 2: Fig. S1. Experimental design of the transcriptomic analysis on Hyla orientalis. Fig. S2. Contigs expression and population structure analysis. a. PCA on RNAseq expression data. b. DAPC on genetic variants obtained from the 87 individuals. c. Results of the K-mean clustering of individuals based on genetic polymorphisms. d. Admixture analysis made on the SNP from the 87 individuals to assess ancestry coefficient. Fig. S3. Bootstrap analysis after hierarchical clustering of 5,735 contigs expressed in 87 individuals. AU (Approximately Unbiased) p-value and BP (Bootstrap Probability) value are represented at each node in red and green respectively. AU p-value (computed by multiscale bootstrap resampling) is a good approximation of unbiased p-value. Branches with confidence intervals > 90% are highlighted in red. Fig. S4. Comparison of the differential expression analysis (n = 17,323 contigs) using G18, H18 or F18 populations as references. The correlation of the FC obtained by using the different reference sites is indicated in each comparative analysis. The Pearson correlation coefficient rho is indicated at the top. Fig. S5. a. Unsupervised hierarchical clustering of normalised expression of 5,735 contigs differentially expressed in at least one population (using G18 individuals as control) after removing the effects of genetic distances as covariables. The expression levels of contigs (as Row Z-score) are represented as follows: low expression (blue), moderate expression (white) and high expression (red). b. Enrichments of Gene Ontology Biological Process in the different clusters obtained by hierarchical clustering. The numbers in brackets correspond to the total number of contigs in each cluster (bottom, x-axis). Fig. S6. Association between contig expression and individual variables (BCI and ITDR) by WGCNA after adjusting the expression for genetic distances and removing low abundance genes (n = 13,641). Traits associations with module eigengene [file 12915_2023_1659_MOESM2_ESM.pdf]

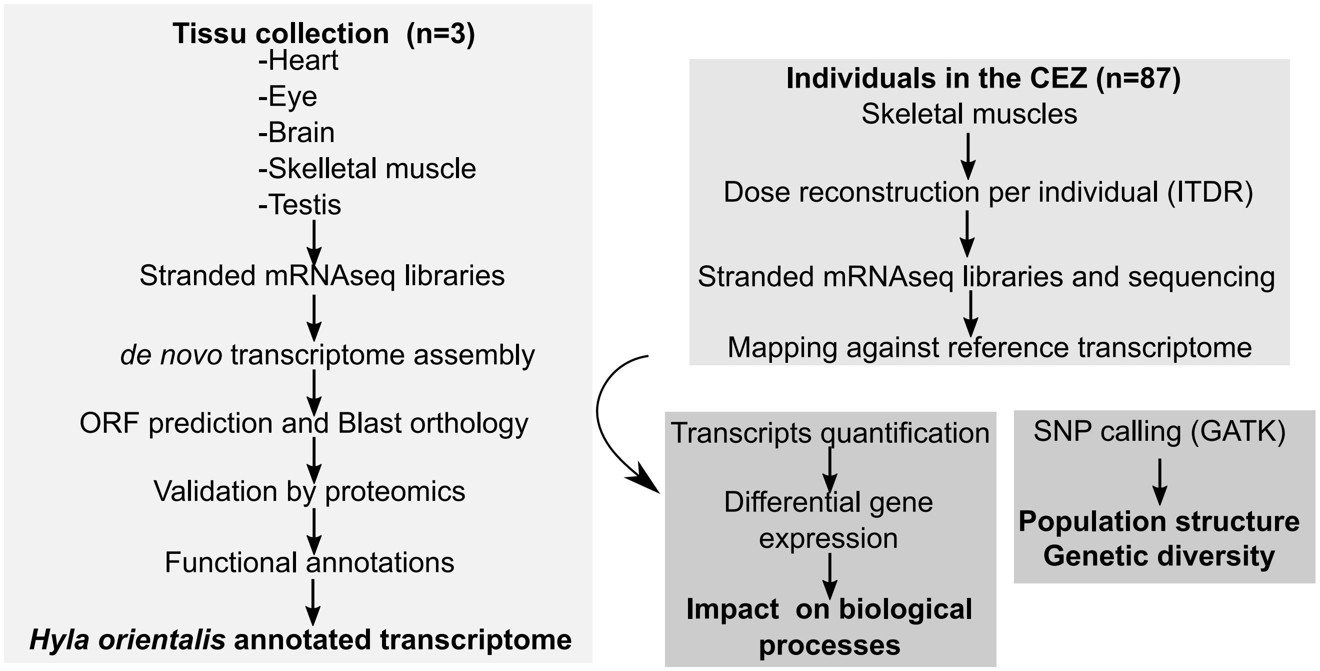

Tissu collection (n=3)
-Heart
-Eye
-Brain
-Skelletal muscle
-Testis
Stranded mRNAseq libraries
de novo transcriptome assembly
ORF prediction and Blast orthology
Validation by proteomics
Functional annotations
Hyla orientalis annotated transcriptome
Individuals in the CEZ (n=87)
Skeletal muscles
Dose reconstruction per individual (ITDR)
Stranded mRNAseq libraries and sequencing
Mapping against reference transcriptome
Transcripts quantification
Differential gene expression
Impact on biological processes
SNP calling (GATK)
Population structure
Genetic diversity

Figure 1

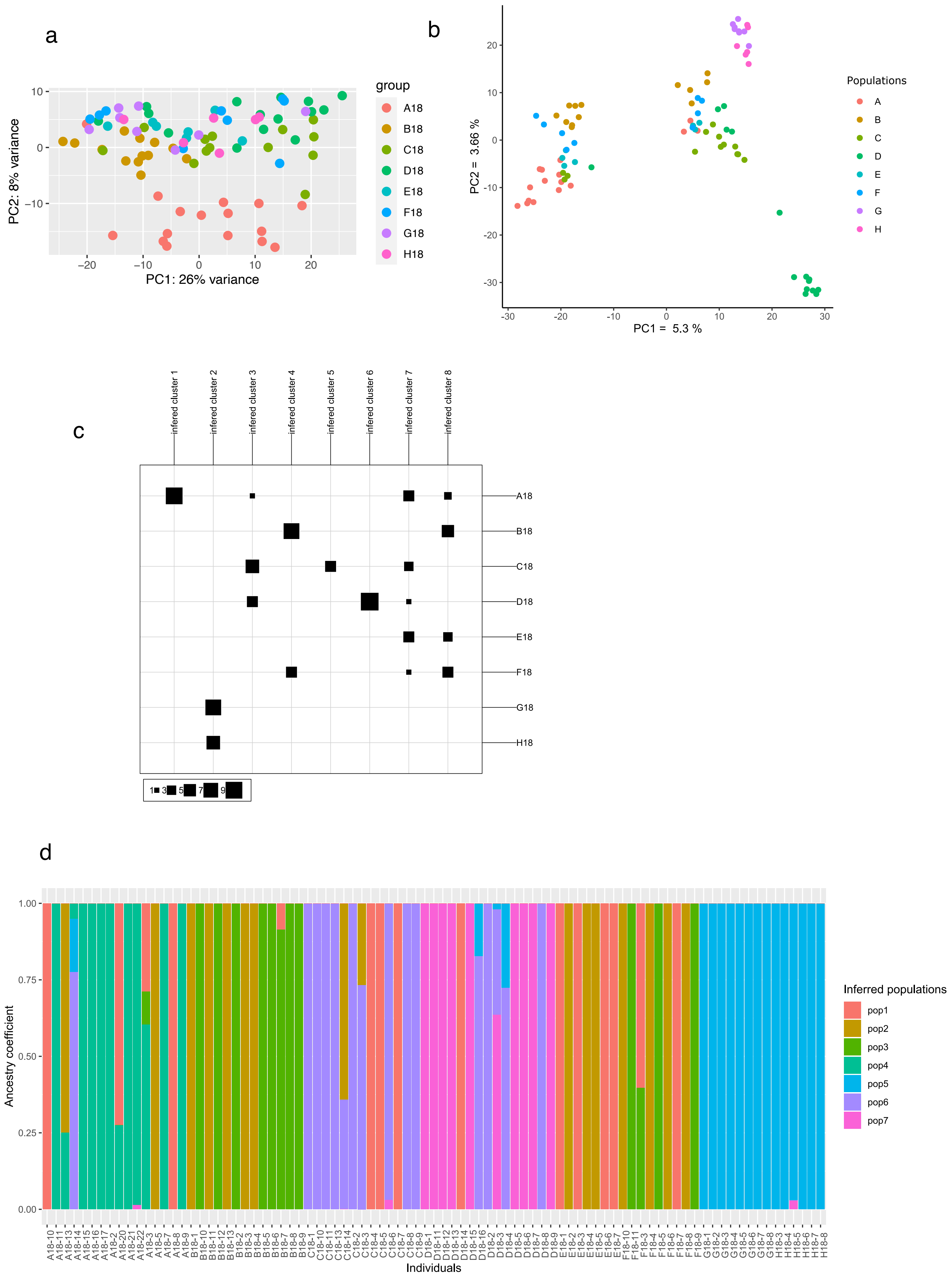
a
PC1: 26% variance
PC2: 8% variance
group
A18
B18
C18
D18
E18
F18
G18
H18
b
PC1 = 5.3 %
PC2 = 3.66 %
Populations
A
B
C
D
E
F
G
H
c
infered cluster 1
infered cluster 2
infered cluster 3
infered cluster 4
infered cluster 5
infered cluster 6
infered cluster 7
infered cluster 8
A18
B18
C18
D18
E18
F18
G18
H18
1 3 5 7 9
d
Ancestry coefficient
Individuals
Inferred populations
pop1
pop2
pop3
pop4
pop5
pop6
pop7

Figure 2

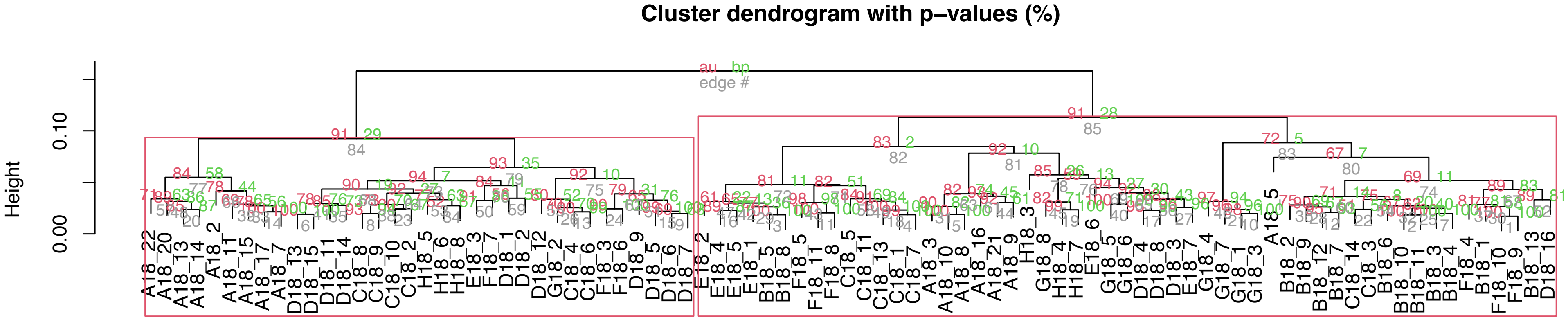

Figure 3

Distance: correlation
Cluster method: complete

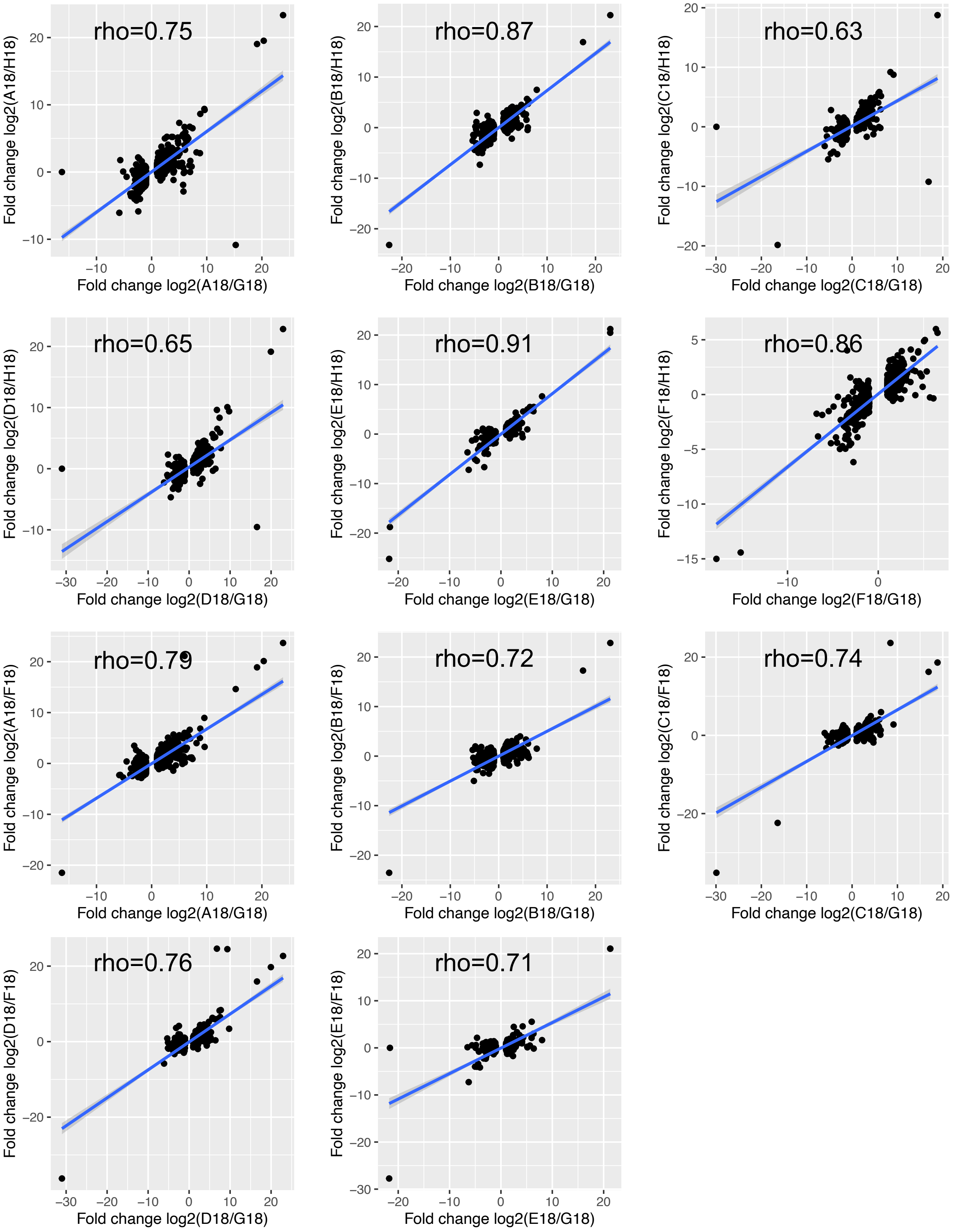
rho=0.75
Fold change log2(A18/H18)
Fold change log2(A18/G18)
rho=0.87
Fold change log2(B18/H18)
Fold change log2(B18/G18)
rho=0.63
Fold change log2(C18/H18)
Fold change log2(C18/G18)
rho=0.65
Fold change log2(D18/H18)
Fold change log2(D18/G18)
rho=0.91
Fold change log2(E18/H18)
Fold change log2(E18/G18)
rho=0.86
Fold change log2(F18/H18)
Fold change log2(F18/G18)
rho=0.79
Fold change log2(A18/F18)
Fold change log2(A18/G18)
rho=0.72
Fold change log2(B18/F18)
Fold change log2(B18/G18)
rho=0.74
Fold change log2(C18/F18)
Fold change log2(C18/G18)
rho=0.76
Fold change log2(D18/F18)
Fold change log2(D18/G18)
rho=0.71
Fold change log2(E18/F18)
Fold change log2(E18/G18)

Figure 4

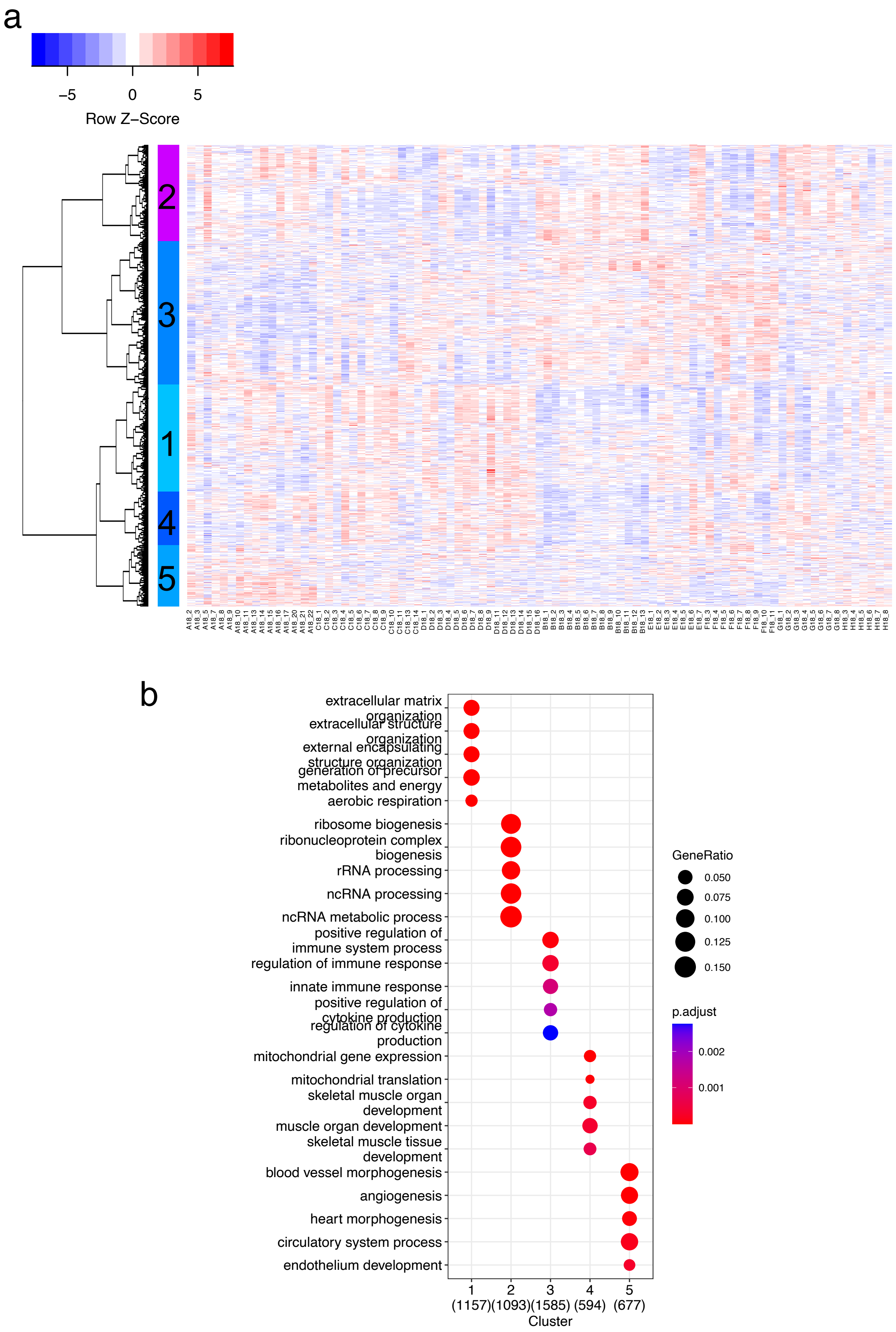
a
−5
0
5
Row Z−Score
2
3
1
4
5
b
extracellular matrix organization
extracellular structure organization
external encapsulating structure organization
generation of precursor metabolites and energy
aerobic respiration
ribosome biogenesis
ribonucleoprotein complex biogenesis
rRNA processing
ncRNA processing
ncRNA metabolic process
positive regulation of immune system process
regulation of immune response
innate immune response
positive regulation of cytokine production
regulation of cytokine production
mitochondrial gene expression
mitochondrial translation
skeletal muscle organ development
muscle organ development
skeletal muscle tissue development
blood vessel morphogenesis
angiogenesis
heart morphogenesis
circulatory system process
endothelium development
1
2
3
4
5
(1157)(1093)(1585) (594) (677)
Cluster
GeneRatio
0.050
0.075
0.100
0.125
0.150
p.adjust
0.002
0.001

Figure 5

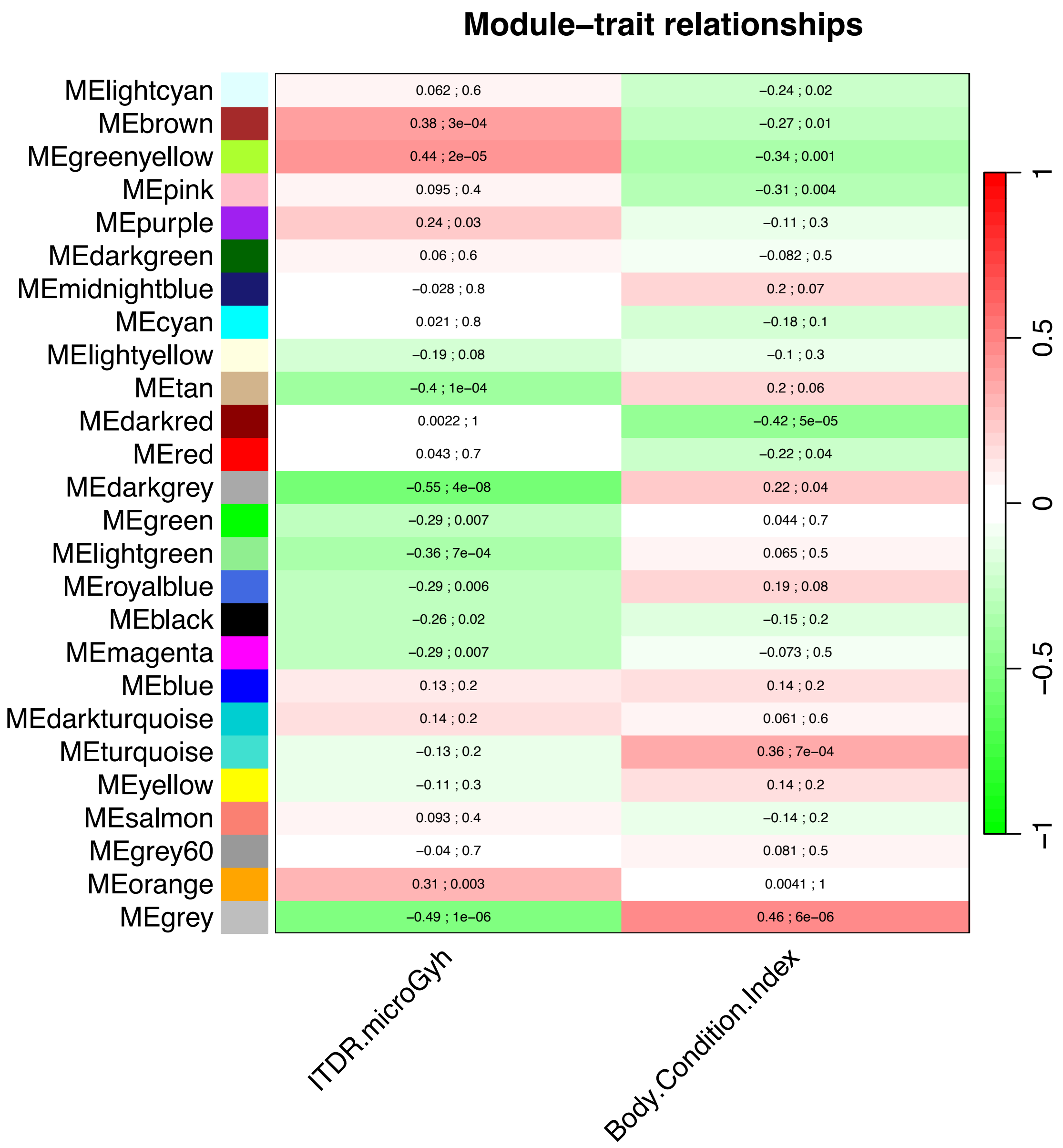
Module–trait relationships
MElightcyan
MEbrown
MEgreenyellow
MEpink
MEpurple
MEdarkgreen
MEmidnightblue
MEcyan
MElightyellow
MEtan
MEdarkred
MEred
MEdarkgrey
MEgreen
MElightgreen
MEroyalblue
MEblack
MEmagenta
MEblue
MEdarkturquoise
MEturquoise
MEyellow
MEsalmon
MEgrey60
MEorange
MEgrey
0.062 ; 0.6
0.38 ; 3e−04
0.44 ; 2e−05
0.095 ; 0.4
0.24 ; 0.03
0.06 ; 0.6
−0.028 ; 0.8
0.021 ; 0.8
−0.19 ; 0.08
−0.4 ; 1e−04
0.0022 ; 1
0.043 ; 0.7
−0.55 ; 4e−08
−0.29 ; 0.007
−0.36 ; 7e−04
−0.29 ; 0.006
−0.26 ; 0.02
−0.29 ; 0.007
0.13 ; 0.2
0.14 ; 0.2
−0.13 ; 0.2
−0.11 ; 0.3
0.093 ; 0.4
−0.04 ; 0.7
0.31 ; 0.003
−0.49 ; 1e−06
−0.24 ; 0.02
−0.27 ; 0.01
−0.34 ; 0.001
−0.31 ; 0.004
−0.11 ; 0.3
−0.082 ; 0.5
0.2 ; 0.07
−0.18 ; 0.1
−0.1 ; 0.3
0.2 ; 0.06
−0.42 ; 5e−05
−0.22 ; 0.04
0.22 ; 0.04
0.044 ; 0.7
0.065 ; 0.5
0.19 ; 0.08
−0.15 ; 0.2
−0.073 ; 0.5
0.14 ; 0.2
0.061 ; 0.6
0.36 ; 7e−04
0.14 ; 0.2
−0.14 ; 0.2
0.081 ; 0.5
0.0041 ; 1
0.46 ; 6e−06
1
0.5
0
−0.5
−1
ITDR.microGyh
Body.Condition.Index

Figure 6
