# Supplementary material for: Population transcriptogenomics highlights impaired metabolism and small population sizes in tree frogs living in the Chernobyl Exclusion Zone
Source: BMC Biol. 2023 Jul 31;21:164. doi: 10.1186/s12915-023-01659-2 (PMC10391870; doi:10.1186/s12915-023-01659-2)
Supplement: Supplementary file 3 — Additional file 3. Supplementary material and methods. [file 12915_2023_1659_MOESM3_ESM.docx]

Additional file 3:

Supplementary Material and Methods

*Generation of the Hyla orientalis reference transcriptome*

Transcriptome assembly was performed by using the combined set of 5.9 billion RNA-Seq paired end-reads from 5 tissues (tibia muscle, heart, eye, brain and testis) obtained from 3 *Hyla orientalis* specimens collected near Slavutich locality. Trinity version 2.8.6 was used for *de novo* transcriptome assembly following best practices (Adam Freedman and Nathan Weeks, Harvard, <https://informatics.fas.harvard.edu>). More specifically, we removed erroneous k-mers with rCorrector and discarded low complexity reads. Adapters and low quality reads were removed with trim_galore v0.6.6 (https://github.com/FelixKrueger/TrimGalore), a wrapper for cutadapt, using the options --length 36 --retain_unpaired --paired -q 10 --stringency 5 -e 0.1 --trim-n. We then identified and removed ribosomal RNA with the SILVA LSU and SSU rRNA database release 132, enriched with *Hyla arborea* and *Hyla orientalis* 12S, 16S and 28S rRNA, by mapping with bowtie2 v2.3.5.1 with the option --very-sensitive-local. Quality controls of reads was re-assessed with fastqc v0.11.9. The resulting reads were finally assembled using Trinity with the option --SS_lib_type RF. Completeness of *Hyla orientalis* transcriptome assembly was assessed by BUSCO analysis. The number of complete transcript was 95.3% (n= 3950).

*Proteomics analysis and proteome database constitution*

Proteins were extracted from muscles of three individuals collected near Slavutich using a potter Elvehjem in RIPA sample buffer (Sigma), purified following the protocol detailed before, and subjected to a short SDSPAGE electrophoretic migration. Gels were incubated with Imperial Protein Stain (Thermo Fisher Scientific) and after few washes with water, protein bands were cut in 5 pieces along their molecular weight and submitted to classical in gel protein digestion. Peptides were analysed using liquid nanochromatography online in front of an Orbitrap Fusion Lumos Tribrid mass spectrometer (Thermo Fisher Scientific) as previously describe. For data processing, we used a proteogenomics cascade strategy. First, the full ORF dataset (391,124 entries) was processed to eliminate redundancy. The resulting ORF dataset was then used as protein database and queried with the MaxQuant version 15.3.8 at 10% FDR. The result of this first query was used to generate a potential protein database limited to 21,619 ORFs corresponding to the most-relevant candidate proteins. Finally, this database was used for the final processing at 1% FDR resulting in more than 6,000 proteins entries identified on a 6 log dynamic range. This high number of proteins entries suggest a high quality of the ORF dataset.

*Validation of transcriptome assembly by proteomics*

A selected set of 17,323 contigs with high confidence similarity was used for validation by proteomics. This set was obtained by selecting contigs coding for peptides with blastx E < 1e-100 against Uniref90, and selecting for the longest assembled contigs when multiple contigs were coding for the same predicted peptide. To compare transcriptomics and proteomics data, assembled contigs expressed in the muscle (tpm > 0.1 in all triplicates) (n= 7,286) were used as a database to analyse the spectra obtained by proteomics. Matches between predicted proteins from the assembled transcripts and the proteomics ORF dataset were considered when the protein was detected in at least one sample (out of a total of three) by proteomics. This resulted in 3,611 contigs encoding peptides (50%) that had their sequence validated by Mass spectrometry spectra.

*Transcript abundance estimation and expression analysis*

Quality filtered and trimmed paired-end reads of the 87 samples were mapped against the *de novo* assembled *Hyla orientalis* transcriptome using Bowtie2 and RSEM via the Trinity utility *align_and_estimate_abundance.pl* and using the options --prep_reference --SS_lib_type RF --est_method RSEM --aln_method bowtie2 --trinity_mode. RSEM results were imported in R with the function *tximport* and analysed for differential expression without filtering on contigs abundance. Comparative analysis to reference sites (G18, H18 and F18) were performed with DESeq2 using q-value < 0.05 (False Discovery Rate) and |fold change| >= 1.5 for significancy on the set of 17,323 contigs. Non-supervised hierarchical expression analysis was performed on variance-stabilized data (vst) using a set of 5,735 contigs differentially expressed significantly in at least one comparative analysis made with *DESeq2* (using G18 as reference) with *hclust* Spearman’s correlation and complete-linkage method for rows (contigs expression). Enrichments of GO and Kyoto Encyclopedia of Genes and Genomes (KEGG) biological processes were made through human orthology (using a gene universe of 11,342 unique Uniprot proteins expressed in the muscle) with the R package *clusterProfiler* using a cut off q-value < 0.05 (fdr). An additional non-supervised hierarchical expression analysis was performed on the vst data (n=5,735) corrected for genetic distances, considered here as a covariate. To compute this covariate variable, we performed a group factor obtained by hierarchical clustering on the genetic distance matrix. The vst data was then corrected for genetic distance by regression using this factor. The resulting matrix of expression was then submitted to hierarchical clustering (without clustering of samples, as requested), and enrichment performed on each cluster, as before. Gene set enrichment analysis was made using the R *clusterProfiler* and *enrichplot* using the three section of GO repository (BP, MF and CC) and using 10000 permutations. ). For analysis of similarity between individual, bootstrap analysis was made with *pvclust* on the set of 5,735 differentially expressed contigs with 1000 permutations, then cophenetic distance at the sample level were extracted from the dendrogram and correlated to ITDR controlling for genetic distance or geographical distance by partial Mantel test using the R package *vegan* and 9999 permutations.

*Dose-response modelling*

Dose-response modelling (for Bench Mark Dose estimation, BMD) of contig expression was performed using *Dromics* 2.4 on vst expression data (n=17,323) and the ITDR of each frog without filtering on contigs abundance. Contigs significantly responding to ITDR were selected using the quadratic trend test with a q-value (FDR) < 0.05 BMD1_SD_ were calculated using the best fit dose-response model (among monotonic Hill, linear and exponential models, and biphasic Gauss-probit and log-Gauss-probit models) on each selected contig and their 95% confidence intervals obtained with 1000 bootstrap iterations. All contigs with defined BMD were kept for GO term enrichment with *ClusterProfiler*.

*Weighted gene co-expression network analysis*

Weighted gene co-expression network analysis (WGCNA) was carried out on vst normalized expression data obtained from *DESeq2* (n=17,323) with *WGCNA* without filtering on contigs abundance. The network dendrogram was built by computing adjacency using signed network, soft threshold power set to 6 (based on approximate scale free typology) and biweighted midcorrelation (bicor). Adjacency were then transform into signed Topological Overlap Matrix to minimize the effects of noise and spurious associations as recommended, using the function TOMSimilarity. Modules of co-expressed contigs were identified by the Dynamic Tree Cut method with the options deepSplit = 2, pamRespectsDendro = FALSE, minClusterSize = 30. Highly similar modules (correlation > 0.75) were merged, as recommended. BCI and ITDR were then associated to the resulting module eigengenes to explore the correlation between gene expression profiles and phenotypic traits using a threshold p-value < 0.001. To adjust for genetic distances we extracted the eigengene from the module correlated best with the ITDR and applied Phylogenetic linear models (pls) to control for genetic distances using the R package *caper*. A second *WGCNA* analysis was made using a matrix of expression filtered for contigs expressed above the 1^st^ quantile of the data in at least 40 individuals (n=13,641). This matrix was then corrected for genetic distances using the R package *lm* and submitted to module detection and enrichment of GO terms as described before.
